# Network meta-analysis on patent foramen ovale: is a stroke or atrial fibrillation worse?

**DOI:** 10.1007/s10072-020-04922-4

**Published:** 2020-11-26

**Authors:** Leonardo Varotto, Gianni Bregolin, Mariemma Paccanaro, Antonella De Boni, Carlo Bonanno, Francesco Perini

**Affiliations:** 1 Department of Cardiology, San Bortolo Hospital, viale Rodolfi 37, 36100 Vicenza, Italy; 2 Department of Prevention, AULSS 8 Berica, via IV Novembre 46, 36100 Vicenza, Italy; 3 Department of Neurology, San Bortolo Hospital, viale Rodolfi 37, 36100 Vicenza, Italy

**Keywords:** Cryptogenic, PFO or patent foramen ovale, Ischemic stroke or cerebrovascular accident, Transient ischemic attack or TIA, Paradoxical embolism

## Abstract

**Objective:**

Systematic reviews suggest that patent foramen ovale closure (PFOc) is performed percutaneously with low complication rates. We did a network meta-analysis (NMA) comparing devices for PFO closures, evaluating safety and efficacy of transcatheter PFOc in preventing neurological events in patients with stroke when compared with medical therapy (MT), and assessing risk of atrial fibrillation (AF).

**Methods:**

We searched 3 databases (MEDLINE, EMBASE, CENTRAL/CCTR) identifying six randomized controlled trials from 2012 until December 2019. We performed a Bayesian NMA; number-needed-to-treat and number-needed-to-harm were derived by applying the estimated odds ratios (ORs). The likelihood of being helped or harmed (LHH) was evaluated to estimate the risk-effectiveness balance.

**Results:**

The 3560 patients allocated to PFOc were less subject to a stroke than patients with MT. The overall ORs of PFOc versus MT were 0.41 with fixed-effects, and 0.22 with random-effects model. NMA proves that PFOc induces AF episodes significantly higher than MT, even when analysis is limited to only new episodes of “serious AF.” LHH (0.68 fixed-effects, 0.79 random-effects) showed that strokes saved are less than cases of AFs added. By considering only serious AF, strokes saved are higher than serious AFs induced by the PFOc (LHH was 3.46 and 4.00 respectively).

**Conclusions:**

NMA supported PFOc in patients with cryptogenic stroke, confirming that devices are better than MT, but increase the risk of AF by over 2/4 times (serious or unserious AF). Considering serious AFs (real risky clinical condition), patients have more advantages in being treated, since LHH is ≥ 3–4.

**Supplementary Information:**

The online version contains supplementary material available at 10.1007/s10072-020-04922-4.

## Introduction

Ischemic stroke is one of the leading causes of death and disability worldwide. Fundamental to the management of stroke patients is the prevention of further ischemic events.

This rule applies especially to patent foramen ovale (PFO)–related stroke in young patients.

Many meta-analyses suggest that PFO closure can be performed percutaneously with generally low complication rates, but have shown that PFO closure increases the risk of atrial fibrillation (AF) [1–5].

Most cases of AF in the randomized controlled trials (RCTs) occurred early after implantation and consisted of a single paroxysmal episode, that resolved spontaneously, medically, or with cardioversion. Only 3.8% of post-closure AF episodes reportedly progress to permanent AF [6, 7].

Many observations have also stated that the new-onset of AF after PFO closure depends on the type of device used and that the Amplatzer device seems to appear better in preventing episodes of AF, when compared to other devices [8–11].

In the six published RCTs [3, 4, 12–16], 11 different devices were used.

We did a network meta-analysis (NMA) by comparing devices, so as to evaluate the safety and efficacy of transcatheter PFO closure in the prevention of recurring ischemic strokes, in patients with cryptogenic stroke when compared with medical therapy (MT). We sought also to systematically assess the incidence and the risk of new-onset AF in the same patients, evaluating the severity of AF episodes, as reported by the authors of the RCTs.

We therefore performed a NMA to investigate device-specific differences and results, to allow a unified and consistent comparison between groups of different devices.

## Methods

### Strategy and selection criteria

This meta-analysis was performed in accordance with the Preferred Reporting Items for Systematic Reviews and Meta-analyses Protocols (PRISMA-P) statement [17] (Supplementary Figure 1, where the databases and keywords used are also listed).

The study protocol was registered with PROSPERO: CRD42020157003.

Data extraction was performed by two independent investigators (LV, CB) and any discrepancies were resolved by a third author (MP). All selected papers were required to be RCTs comparing experimental and different approved transcatheter PFO closure devices with MT. After examining 497 studies, we identified 6 RCTs through a literature search from 2012 until December 31, 2019, that assessed the efficacy of transcatheter PFO closure for secondary prevention of cryptogenic strokes or TIA, compared to MT. The four groups of devices investigated in randomized trials were Amplatzer PFO Occluder (AMP, Abbott, Chicago, IL, USA), STARFlex Septal Occluder (STF, NMT Medical, Boston, MA, USA), Gore HELEX Septal Occluder/Gore Cardioform Septal Occluder (HLX/CF, W.L. Gore and Associates, Flagstaff, AZ, USA), and a group in which a variety of eleven devices were used during the CLOSE trial [3] (including 238 patients allocated to PFO closure—MIXED devices—, of which 138 with AMP).

### Data collection

We extracted information on study design, outcomes, characteristics of patients, length of follow-up, and components of methodological quality, including concealment of allocation, independent event adjudication, and analysis according to the intention-to-treat principle [18–20]. If multiple reports were available for one trial, we used information of all reports for data extraction, but extracted outcome data only once, based on the intention-to-treat principle and the longest follow-up available. The primary outcome measures of this analysis were recurrences of ischemic stroke. Secondary endpoints included new-onset AF. With the exception of the STF device that had a significantly lower procedural success rate and more pronounced new-onset AFs, we evaluated the remaining devices similarly, in relation to demonstration of a high and comparable frequency of success or risk of new-onset AF.

The cases of new-onset AF have been considered “serious” or because defined by the authors as such or because they were persistent. Late-onset AF was defined as AF occurring > 45 days post-PFO closure. For a correct comparison of the data, we reported all AFs and not only those defined as “serious”, published in RESPECT [15]. The data used for AMP were derived from the new Instruction For Use relating to the January 2018 device, updated by St. Jude Medical in order to collect the extended follow-up of the RESPECT study (data equal to those used to obtain the 2016 FDA approval).

The data were double-checked and the consensus reached to avoid disagreement. We contacted the authors of one RCT for additional data [9].

### Risk of bias

We assessed risk of bias in duplicate using a modified Cochrane Collaboration tool [21]. For each of the seven risk dimensions analyzed, we assigned a low, a moderate, or a high risk of bias to the studies. Reviewers resolved conflicts through discussion. The overall internal validity was considered a whole at “low risk of bias” (Supplementary Figure 2).

### Statistical analysis

To compare the effects of alternative PFO closure with devices versus MT, for ischemic stroke (primary outcome) and recurrent AF (secondary outcomes), we conducted a NMA of RCTs within a Bayesian hierarchical fixed and random-effect framework with non-informative priors to derive the posterior distribution [22]. We used pooled ORs with credible intervals (Cr.Is) 95% as statistical indicator.

Analyses were done in NetMetaXL 1.6. [23] and WinBUGS 1.4. These programs provide the summary tables, the network diagram, the forest plot, the league table, the ranking list with SUCRA (Surface Under the Cumulative RAnking curve) indicator, and heterogeneity. We evaluated convergence using the Brooks-Gelman-Rubin method.

We also assessed inconsistency by comparing deviation residues and statistics of deviance information criteria. Inconsistency plots assessing network inconsistencies between direct and indirect estimates showed a low possibility that the inconsistencies may significantly affect the network meta-analysis results (Supplementary Figure 3 and Supplementary Figure 4).

Neither the heterogeneity index, nor the qualitative/visual analysis of the funnel plot produced in the exploratory phase, nor much less the analysis of inconsistency has raised the suspicion that the trials cannot be compared and analyzed jointly in order to produce a synthesis result.

Numbers-needed-to-treat (NNT) and number-needed-to-harm (NNH) were derived by transforming the estimated odds ratios (ORs). The likelihood of being helped or harmed (LHH) was evaluated to estimate the risk-effectiveness balance [24].

## Results

Four hundred and ninety-seven records were found by searching databases and trial registers until the end December 2019. Six randomized, multicenter, controlled open-label trials (with blinded adjudication of outcome events) were identified (Supplementary Table 1).

Accordingly, the six trials [3, 4, 12–16] and 3560 patients (the mean age was 45.4 to 46.2 years) with cryptogenic stroke and PFO were included in the review and NMA, with four treatment groups with devices versus MT, to study the efficacy of PFO closure for secondary prevention. The mean follow-up period of all 6 RCTs was 2 to 5.9 years. Baseline characteristics and events in the RCTs are reported in Supplementary Table 2.

**Fig. 1:**
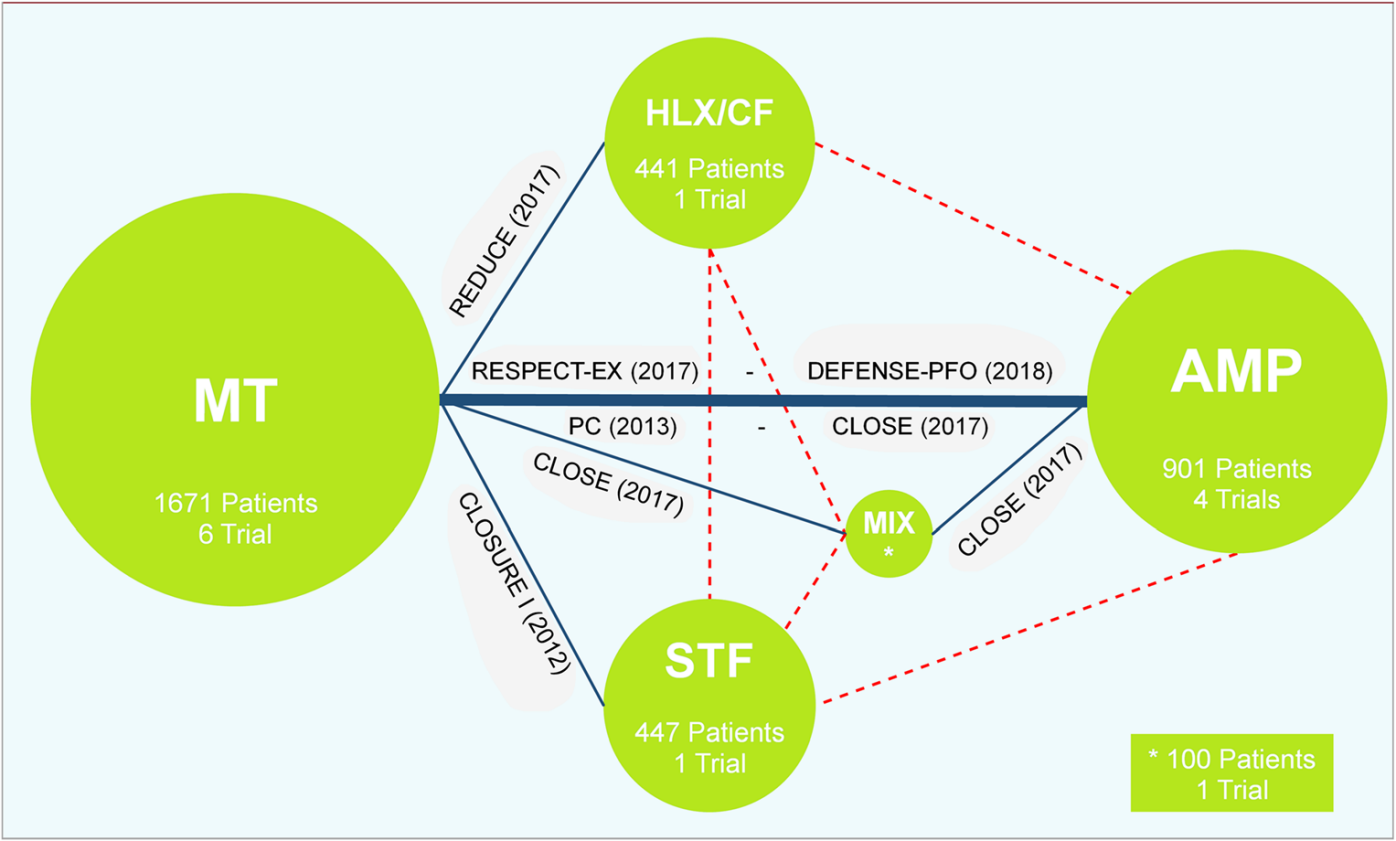
Network of comparisons included into the analyses. Legend: The size of every green circle is proportional to the number of randomized patients in the different trials. The width of the blue line corresponds to the number of trials, and it indicates a direct comparison. The dashed red lines indicate the comparison relations «indirect» through the network meta-analysis. MT, medical therapy; HLX/CF, Helex/Cardioform; MIX, mixed devices; AMP, Amplatzer; STF, Starflex

Three trials compared PFO closure with AMP versus MT (group AMP), one PFO closure with STF versus MT (group STF), and one PFO closure with HLX/CF versus MT (group HLX/CF). The CLOSE Trial used 11 devices, and 238 patients were assigned to the PFO closure versus the antiplatelet-only therapy. We have created a subgroup by extrapolating 138 patients treated with three types of AMP (Septal Occluder, Cribriform and Atrial Septal Device) during stroke analysis and moved them to the AMP group, which concerns a total of 901 patients included in 4 trials. The other 100 patients for CLOSE trial remained in a group with 8 different devices (group MIXED devices) and were compared with MT. Figure 1 shows the network of evidence.

**Fig. 2:**
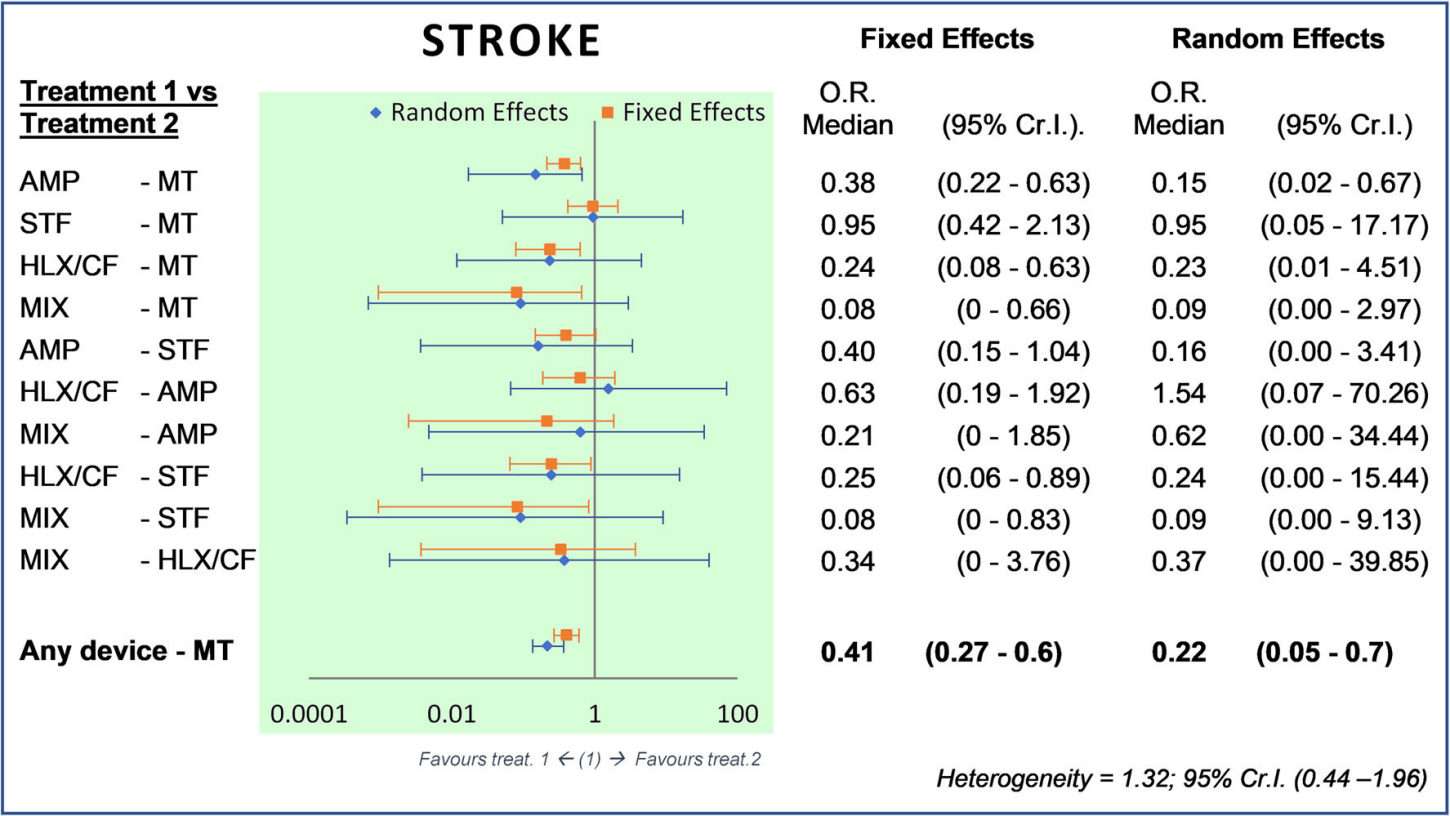
Bayesian comparison between treatments: stroke’s events. Legend: Forest plot and odds ratio estimated with 95% credible interval, fixed, and random model. The AMP’s relevance is 19/901 = 2.1% (events/patients); STF 12/447 = 2.7%; HLX/CF 6/441 = 1.4%; MIX 0/100 = 0%; MT 77/1671 = 4.6%. MT, medical therapy; HLX/CF, Helex/Cardioform; MIX, mixed devices; AMP, Amplatzer; STF, Starflex; Cr.I., credible interval; OR, odds ratio

Patients allocated to PFO closure were significantly less likely to experience a stroke compared with patients allocated to MT. From NMA, the ORs for any device versus MT were 0.41 (Cr.I. 95%, 0.27–0.60) with fixed-effects model and 0.22 (Cr.I. 95%, 0.05–0.70) with random-effects model (Fig. 2).

The patients allocated to the AMP group had similar risk as patients with HLX/CF group or with group of 8 mixed devices (MIX). The AMP device proves to be more variable in terms of results (between fixed and random effects) as regards its effectiveness in containing further strokes, with results similar to the HLX/CF device, suggesting their equality.

**Fig. 3:**
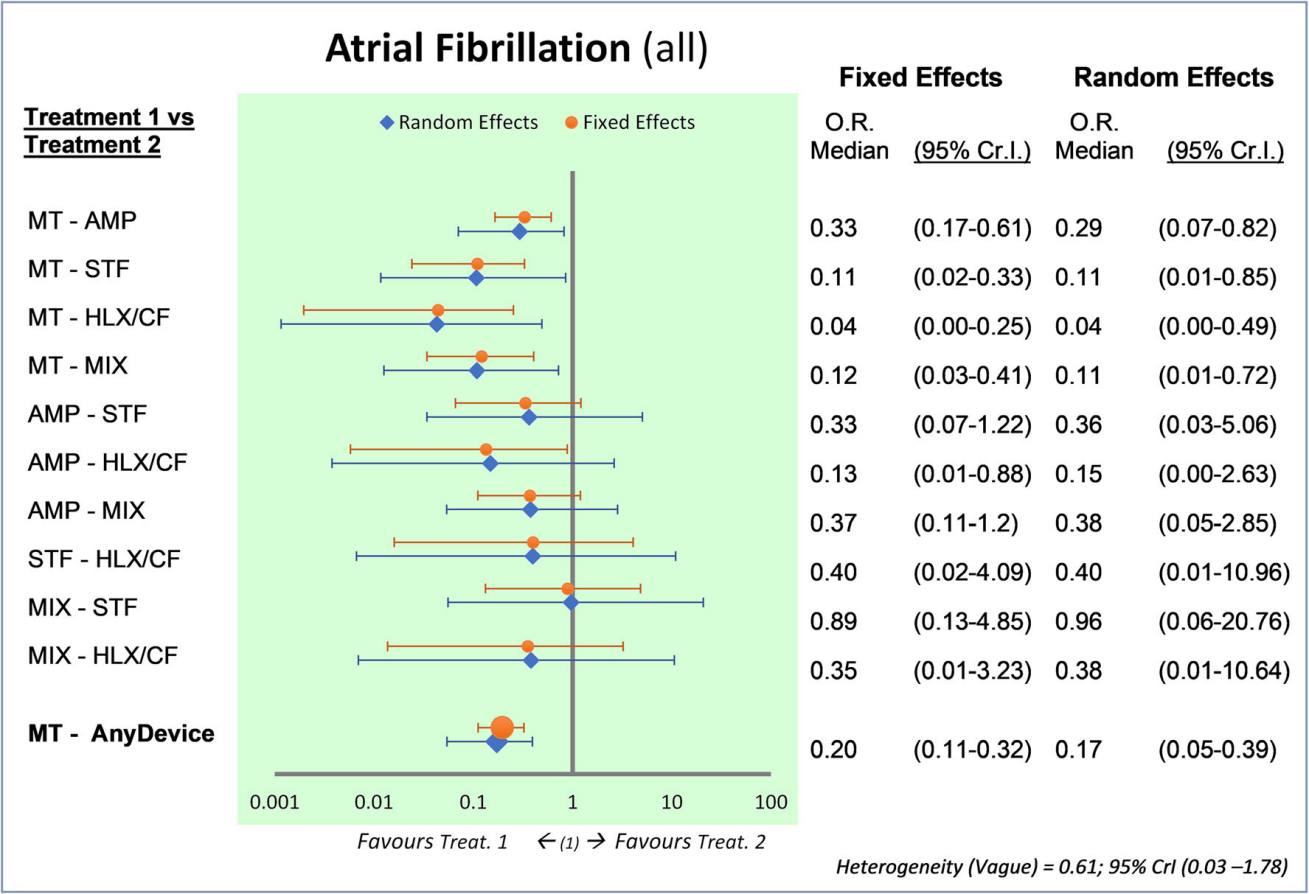
Bayesian comparison between treatments: atrial fibrillation (all events). Legend: Forest plot and odds ratio estimated with 95% credible interval, fixed, and random model. The AMP’s relevance is 36/901 = 4% (events/patients); STF 23/447 = 5.1%; HLX/CF 29/441 = 6.6%; MIX 7/100 = 7%; MT 17/1671 = 1%. AF, atrial fibrillation; MT, medical therapy; HLX/CF, Helex/Cardioform; MIX, mixed devices; AMP, Amplatzer; STF, Starflex; Cr.I., credible interval; OR, odds ratio

However, in our NMA, PFO closure increased the risk of new-onset AF. The NMA demonstrated that MT induces a number of AF episodes significantly less than all the devices (OR 0.20 fixed-effects, 95% Cr.I., 0.11–0.32; OR 0.17 random-effects, Cr.I. 95% 0.05–0.39) (Fig. 3).

The risk of causing an AF is just over 4 times higher than in MT (risk ratio = 4.10, 95% C.I. 2.43–6.92).

In favor of the AMP, there would be a greater containment of cases of AF vs MT, compared to HLX/CF, but not the superiority of a device compared to this or another device when we use the random-effect model.

**Fig. 4:**
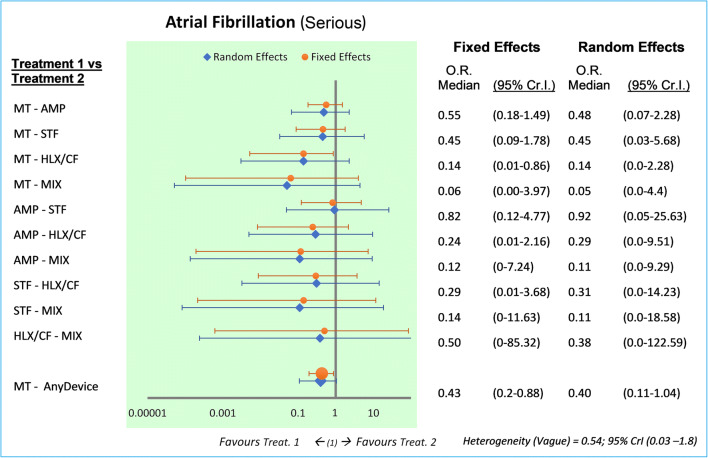
Bayesian comparison between treatments: atrial fibrillation (serious events). Legend: Forest plot and odds ratio estimated with 95% credible interval, fixed, and random model. The AMP’s relevance is 10/901 = 1.1% (events/patients); STF 6/447 = 1.3%; HLX/CF 10/441 = 2.3%; MIX 0/100 = 0%; MT 10/1671 = 0.6%. AF, atrial fibrillation; MT, medical therapy; HLX/CF, Helex/Cardioform; MIX, mixed devices; AMP, Amplatzer; STF, Starflex; Cr.I., credible interval; OR, odds ratio

The same trend is obtained if we consider only the episodes of AF defined as serious (OR 0.43 fixed-effects, 95% Cr.I., 0.2–0.88; OR 0.40 random-effects, Cr.I. 95% 0.11–1.04), but the risk of causing an AF with PFO closure versus MT is halved (risk ratio = 2.26, 95% C.I. 1.1–4.65) (Fig. 4).

The estimated NNT to prevent one stroke for PFO closure compared with MT was 37 (Cr.I. 95%, 30–57) with fixed-effects model and 28 (Cr.I. 95%, 23–75) with random-effects model; the NNH to cause one case of new-onset AF for PFO closure versus MT was 25 (95% Cr.I. 14–49) with fixed-effects model and 22 (Cr.I. 95%, 7–66) with random-effects model (Table 1).

**Table 1:** Measures of effectiveness and undesirability of PFO closure versus medical therapy

Treatment/device		NNT - stroke	NHH - all events Atrial fibrillation	NHH - serious Atrial fibrillation
		Median value (95% Cr.I.)	Median value (95% Cr.I.)	Median value (95% Cr.I.)
Fixed effects approach	AMP	35 (28–61)	49 (21–156)	210 (39–∞)
	STF	411 (38–∞)	13 (3–49)	141 (17–∞)
	HLX/CF	29 (24–59)	6 (1–35)	28 (2–∞)
	MIX	22 (22–42)	15 (5–69)	12 (1–∞)
	Total	37 (30–57)	25 (14–49)	128 (42–1185)
Random-effects approach	AMP	26 (22–67)	42 (9–463)	156 (13–∞)
	STF	489 (23–∞)	13 (2–575)	137 (7–∞)
	HLX/CF	29 (22–∞)	5 (1–97)	28 (2–∞)
	MIX	22 (22–∞)	13 (2–258)	10 (1–∞)
	Total	28 (23–75)	22 (7–66)	112 (21–∞)

In the table are shown the median value of NNH (number-needed-to-harm) and NNT (number-needed-to-treat) for fixed and random approach, as measures of clinical outcomes in the PFO closure versus medical therapy. The NNH value is calculated both for all atrial fibrillation events and for serious events only

*AMP*, Amplatzer; *STF*, Starflex; *HLX/CF*, Helex/Cardioform; *MIX*, mixed devices; *PERR*, patient-expected event rate; *OR*, odds ratio

NNT = (1-(PEER*(1-OR))) / ((1-PEER)*(PEER)*(1-OR))

NNH = ((PEER*(OR-1)) + 1) / (PEER*(OR-1)*(1-PEER))

**Fig. 5:**
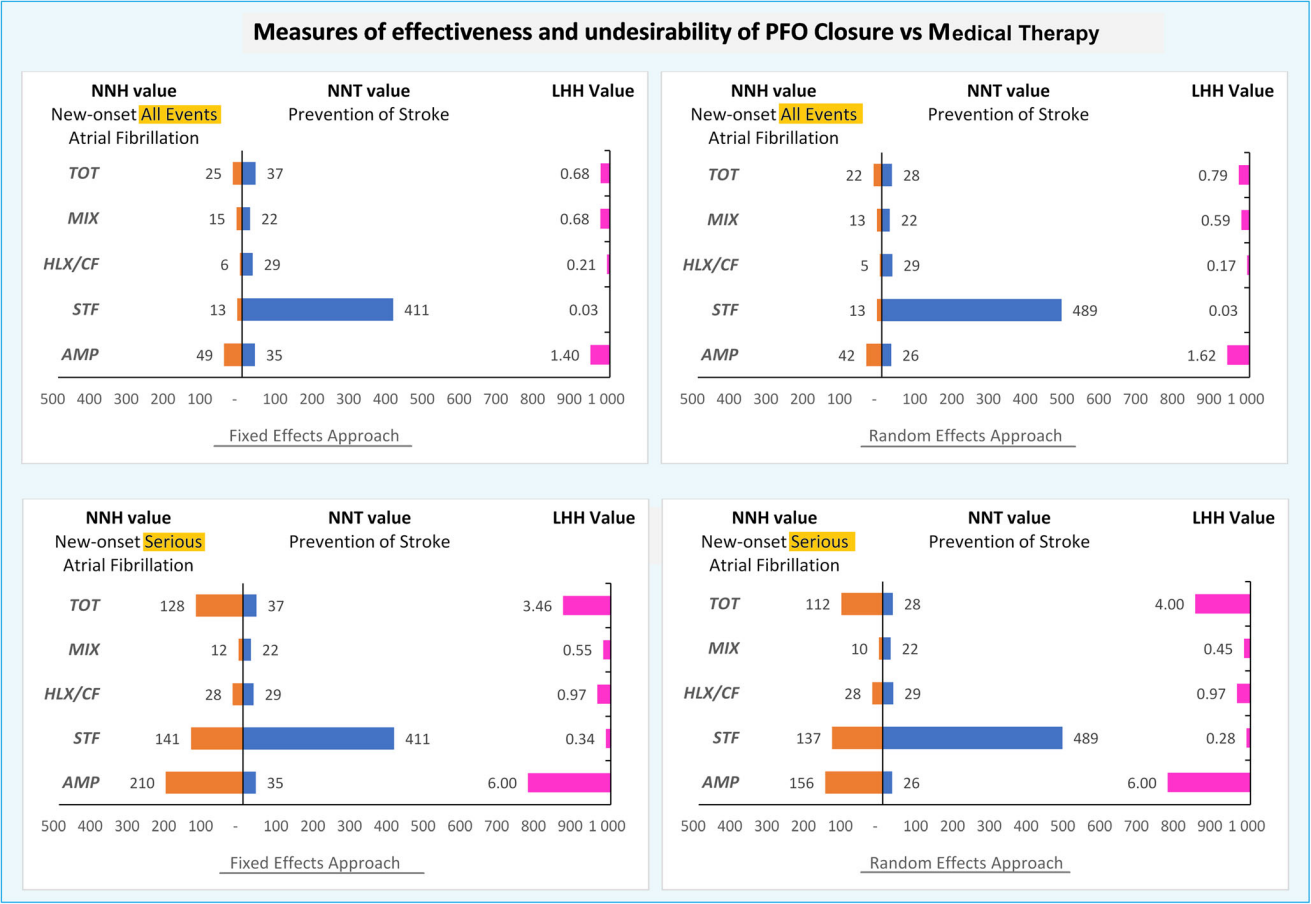
Measures of effectiveness and undesirability of PFO closure versus MT therapy. Legend: In the four graphs are shown the median value of NNH (number-needed-to-harm), NNT (number-needed-to-treat), and its ratio LHH (likelihood of being helped or harmed), for fixed and random approach, as measures of clinical outcomes in the PFO closure versus medical therapy. In one case, all the events of atrial fibrillation (AF) are examined; in the other, only serious AF. AMP, Amplatzer; HLX/CF, Helex/Cardioform; MIX, mixed devices; STF, Starflex, TOT, total value from combined devices

The LHH was 0.68 and 0.79, respectively, and showed that the number of strokes saved is less than that of AF added. If we calculate this parameter considering only the serious AF, the number of strokes saved is higher than that of serious AF induced by the PFO closure (LHH was 3.46 and 4.00 respectively) (Fig. 5).

We have performed a summary comparison between the two most used devices through a bidimensional analysis.

**Fig. 6:**
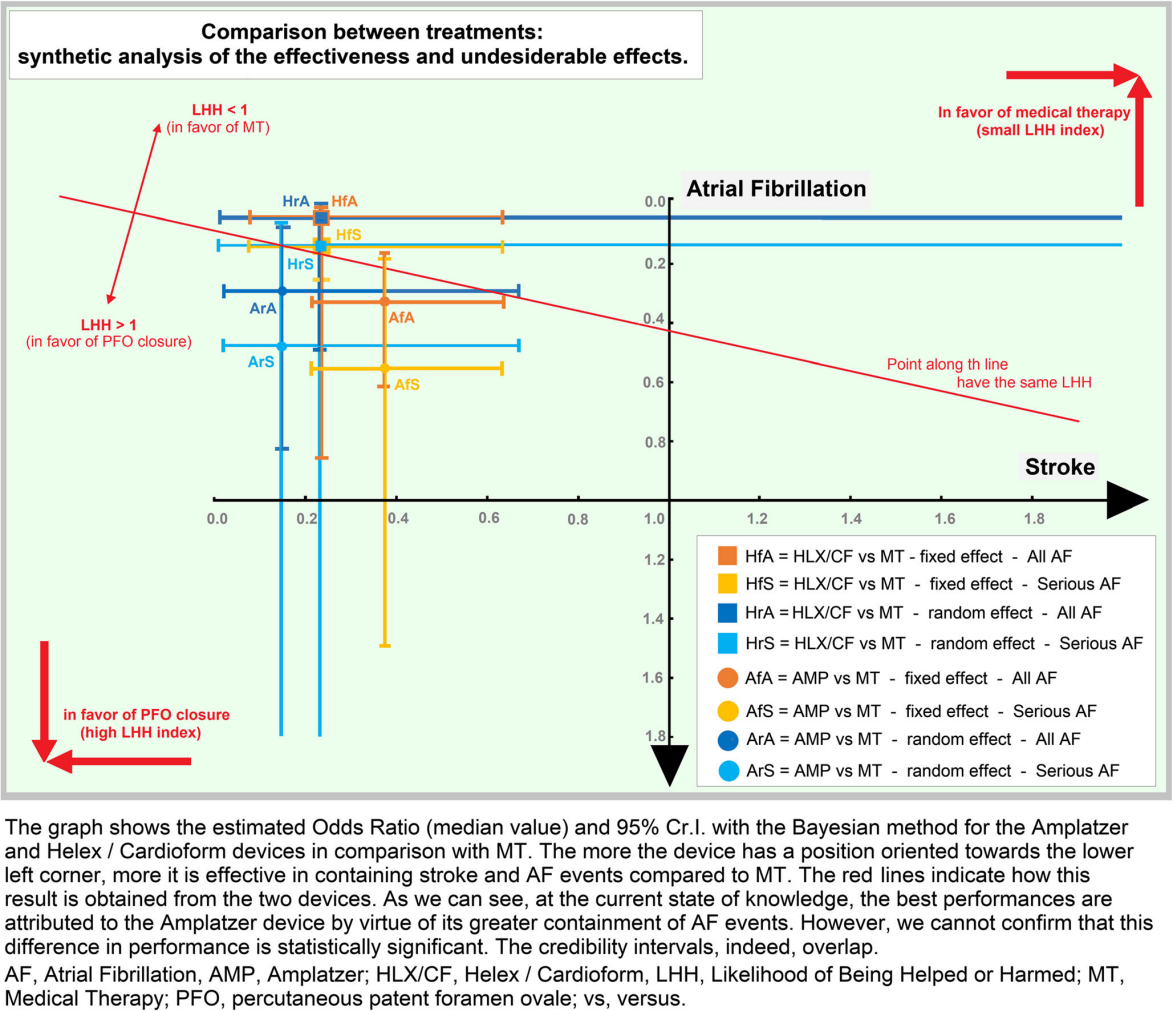
Bidimensional analysis of comparisons between treatments, atrial fibrillation, and stroke. Legend: The graph shows the estimated odds ratio (median value) and 95% Cr.I. with the Bayesian method for the Amplatzer and Helex/Cardioform devices in comparison with MT. The more the device has a position oriented towards the lower left corner, the more it is effective in containing stroke and AF events compared to MT. The red dotted lines indicate how this result is obtained from the two devices. As we can see, at the current state of knowledge, the best performances are attributed to the Amplatzer device by virtue of its greater containment of AF events. However, we cannot confirm that this difference in performance is statistically significant. The credibility intervals, indeed, overlap. AF, atrial fibrillation; AMP, Amplatzer; HLX/CF, Helex/Cardioform; LHH, likelihood of being helped or harmed; MT, medical therapy; PFO, percutaneous patent foramen ovale; vs, versus

In order to visualize the performances of the two main devices in use relating to the two significant events (stroke and AF), a two-dimensional graph has been built which highlights once again that (1) both devices are not substantially different from each other (they remain very close to each other in the fourth quadrant); (2) AMP has better joint median performances (considering both aspect of AF and of stroke), but not for this statistical difference with respect to HLX/CF; (3) the better performance of AMP is mainly linked to the greater containment of AF events, and the result is slightly amplified when the method of analysis adopted is that of random effects; and (4) this performance remains of the same proportion when analyzing serious AF (Fig. 6).

## Discussion

A recent meta-analysis [7] shows that, among the new-onset AFs following percutaneous PFO closure, only < 25% of new-onset AFs become persistent. These AFs could either be primary AFs or device-related AFs, called “therapeutic failure.” The pooled incidence of stroke presumably caused by device-associated AF in the PFO closure group was 0.1%.

Catheter manipulation and wires crossing through the left atrium during closure device deployment can trigger AF.

Some cases of reduction in the incidence of AF with the closure of the foramen ovale have been described [25] (probably due to the reduction of the atrial vulnerability to arrhythmias of the hypermobile interatrial septum, especially when associated with an atrial septal aneurysm [26], or to the decrease of the mechanical stretching of the atrium due to the presence of intermittent interatrial shunting [27, 28]). However, as explained [5], the stretch or septal irritation during and after device deployment can determine a further clinical risk for the patient, which should not be underestimated.

In this work, we wanted to try to reach an effective shared decision-making parameter, proposing a patient-centered measure, i.e., evaluating the probability that a patient could be either helped or harmed by the intervention of percutaneous closure of the foramen ovale. This leaves the discussion open to the evaluation of the usefulness of this procedure and stimulates further investigations.

Apparently, from the clinical point of view, the impact of a stroke compared to a new onset of atrial fibrillation is not the same, but the patient must know by informed consent what may happen and know above all that decision-making processes are developed at the bedside to identify the best therapeutic decisions. The statistical tools are there and have helped us to express the useful and harmful effects of the percutaneous treatment and modify the statements according to the patient’s wishes.

From our NMA, the incidence of AF with devices currently on the market is slightly different, but not significantly different. While we have a duty to analyze well the possible presence of a primary AF with adequate pathways and according to standardized protocols [29], without having to hurry to perform a percutaneous PFO closure, we must also consider that the onset of a device-related AF should not be classified as “therapeutic failure,” at least for devices currently in use and with the data in our possession. Furthermore, if the new (practically absent) ischemic events caused by serious AF could be deduced from the benefit of preventing ischemic events with percutaneous closure of the foramen ovale, AF after closure of the foramen ovale could be regarded as a non-event.

There are some limitations in our NMA. Because the studies analyzed in our NMA defined cryptogenic stroke after excluding primary AF based on short-term conventional monitoring or repeated electrocardiograms, our NMA may also have included some cardioembolic strokes related to not yet fully developed methods or insufficient investigations. Therefore, some AF, which appeared after implanting the device, may not be of first/new-onset.

However, the overall incidence of follow-up AF in the included trials of our NMA was less than previously reported [30]. A precise and careful algorithm for diagnostic workup of cryptogenic left circulation thromboembolism in the next randomized studies (with the aid of loop recorder or patch-based and water-resistant cardiac patch rhythm monitor) [29, 31], associated with better devices and refinement of implantation techniques perhaps will provide us with more precise answers. In this way, the true onset of atrial arrhythmia after implantation of the device could be more clearly evaluated and highlighted, even in asymptomatic patients.

In addition, data could be analyzed in terms of the influence of the age of patients presenting with post-procedural AF, since the importance and risk of AF arising in patients under 40 years old is not the same as that of AF occurring over 40 years.

Further randomized studies seem may not be easy, but certainly in general clinical practice, after having well excluded the cases of primary AF and after PFO closure, it will probably be necessary once again to define and differentiate all AFs from those that are persistent or defined as serious. In fact, the clinical significance of excluding the episodes of transient AF device-related, maintaining only the serious AFs, modifies clearly the risk-benefit balance for the patient.

## Conclusions

Our NMA provides evidence in favor of PFO closure with all the devices used in young patients with cryptogenic stroke and PFO. We can presently conclude that these devices are better than MT, but increase the risk of AF by over 4 times if we consider all cases of AFs and only 2 times with serious AF. We cannot state that one device is better than the rest (AMP has the best performance but is not statistically superior to HLX/C) to reduce AF episodes in the follow-up. Considering serious AFs (real risky clinical condition), patients have more advantages in being treated than in not being, since LHH is ≥ 3–4. Which then is the lesser of two evils?

## Supplementary Information

ESM 1 (PNG 11732 kb)

High resolution image (TIF 2249 kb)

ESM 2 (PNG 7661 kb)

High resolution image (TIF 1902 kb)

ESM 3 (PNG 7928 kb)

High resolution image (TIF 771 kb)

ESM 4 (PNG 14347 kb)

High resolution image (TIF 1431 kb)

ESM 5 (DOCX 18 kb)

ESM 6 (DOCX 25 kb)
