# Supplementary figures and images for: Network meta-analysis on patent foramen ovale: is a stroke or atrial fibrillation worse?
Source: Neurol Sci. 2020 Nov 26;42(1):101–9. doi: 10.1007/s10072-020-04922-4 (PMC7819966; doi:10.1007/s10072-020-04922-4)

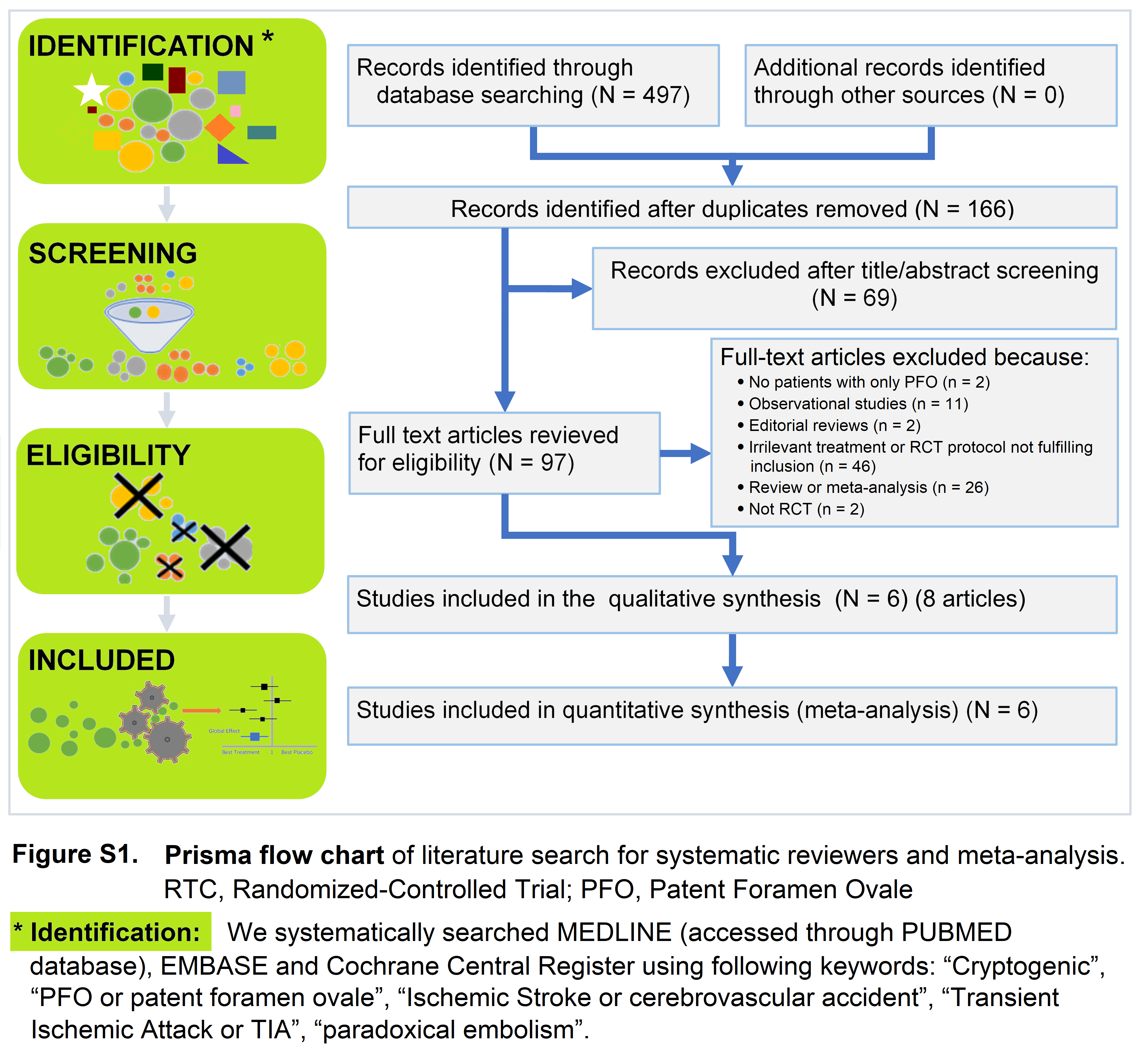

Supplement: Supplementary file 2 — High resolution image (TIF 2249 kb) [file 10072_2020_4922_MOESM1_ESM.tif]

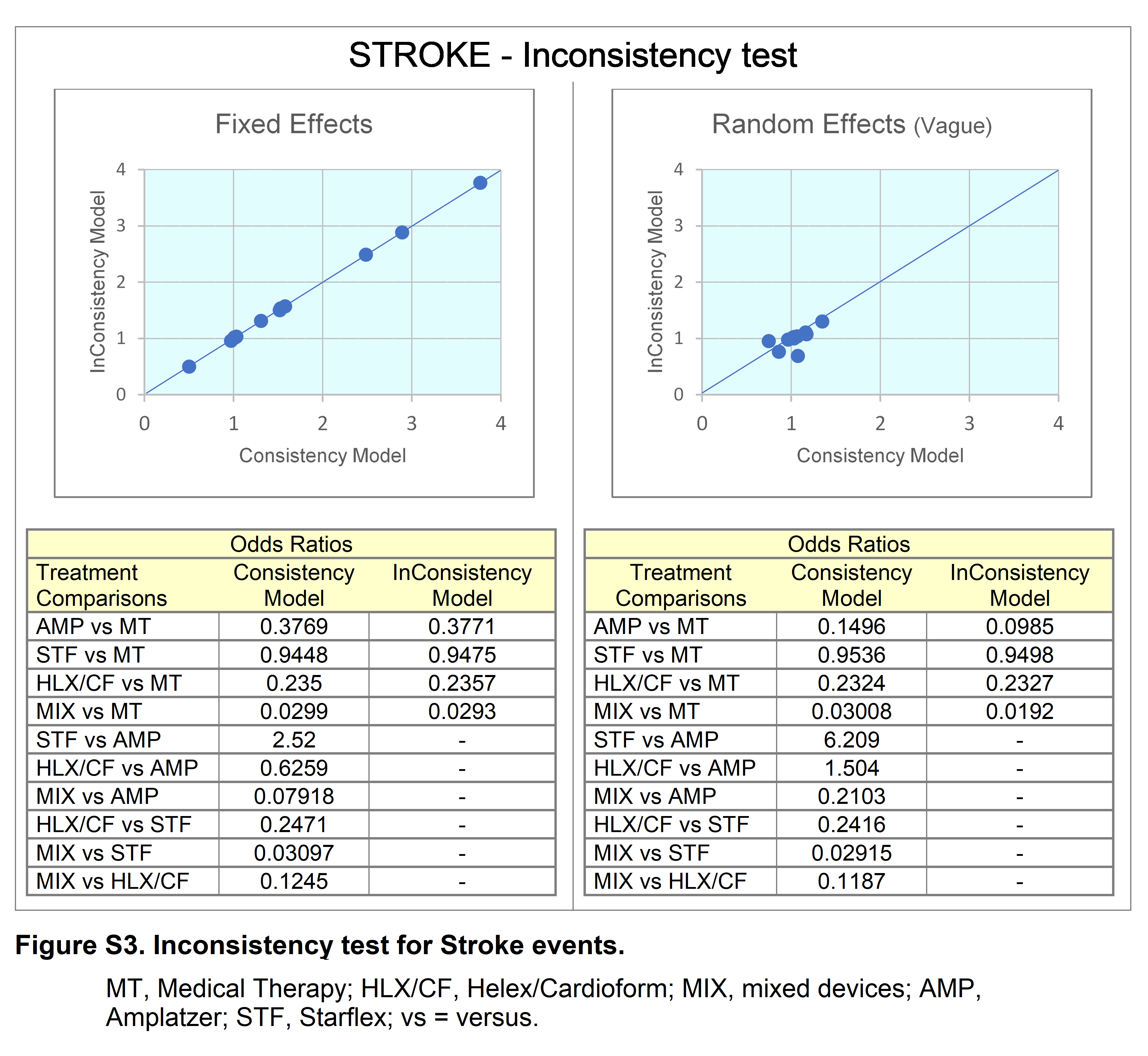

Supplement: Supplementary file 6 — High resolution image (TIF 771 kb) [file 10072_2020_4922_MOESM3_ESM.tif]

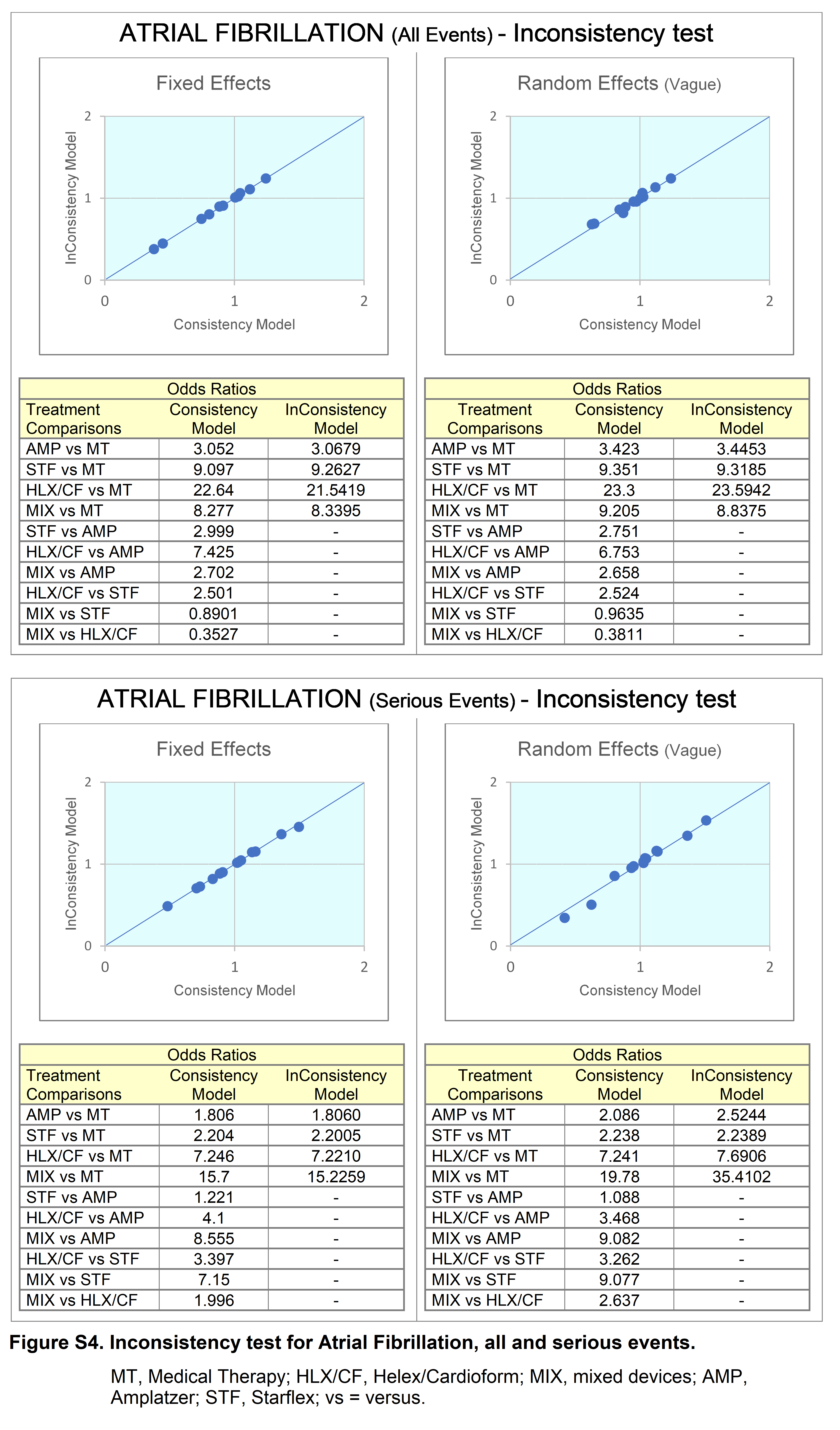

Supplement: Supplementary file 8 — High resolution image (TIF 1431 kb) [file 10072_2020_4922_MOESM4_ESM.tif]
