# Supplementary material for: Network meta-analysis on patent foramen ovale: is a stroke or atrial fibrillation worse?
Source: Neurol Sci. 2020 Nov 26;42(1):101–9. doi: 10.1007/s10072-020-04922-4 (PMC7819966; doi:10.1007/s10072-020-04922-4)
Supplement: Supplementary file 9 — (DOCX 18 kb) [file 10072_2020_4922_MOESM5_ESM.docx]

**Table S1.** Descriptive Summary of the Design of Randomized Clinical Trials included in the NMA

|  | **Closure I** ^13^ | **PC Trial** ^14^ | **RESPECT** ^15,16^ | **REDUCE** ^4^ | **CLOSE** ^3^ | **DEFENSE-PFO** ^17^ |
| --- | --- | --- | --- | --- | --- | --- |
| Pubblication Year | 2012 | 2013 | 2013, 2017 | 2017 | 2017 | 2018 |
| Study Design | Prospective, randomized, blinded adjudication of outcome events  1:1 | Prospective, randomized, blinded adjudication of outcome events  1:1 | Prospective, randomized, blinded adjudication of outcome events  1:1 | Prospective, randomized, blinded adjudication of outcome events  2:1 | Prospective, randomized, blinded adjudication of outcome events  1:1:1 | Prospective, randomized  1:1 |
| Sample Size | N=909 (447;462) | N=414 (204;210) | N=980 (499;981) | N=664 (441;223) | N=473 (238;235) | N=120 (60;60) |
| Inclusion Criteria | - Age 18 to 60 y - Cryptogenic IS or TIA ≤6 months - TEE-verified PFO (with a bubble study showing right-to-left shunting at the atrial level during a Valsalva maneuver) | - Age <60 y - Cryptogenic IS or TIA or peripheral embolism; radiologically verified ischemic lesion mandatory - TEE-verified PFO | - Age 18 to 60 y - Cryptogenic IS ≤270 d - TEE-verified PFO | - Age 18 to 59 y - Cryptogenic IS ≤180 d - TEE-verified PFO | - Age 18 to 60 y - Cryptogenic IS ≤6 months - TEE-verified PFO with large interatrial shunt (<30 microbubbles) or ASA (septum primum excursion >10 mm) | - Age 18 to 80 y - Cryptogenic IS ≤6 months - TEE-verified PFO with atrial septum hypermobility (excursion >10 mm) or ASA (excursion >15 mm) or large PFO size (≥2 mm) |
| Exclusion Criteria | - Potential cause of embolic stroke or TIA other than PFO - Large redundant ASA that cannot be covered by the STARFlex device - Baseline Modified Rankin Score of 3 or greater - Thrombus in or occlusion of the venous lumen between the femoral vein access site and the right atrium | - Identifiable cause for thromboembolic event other than PFO - Contraindication to medical therapy - Follow-up over the next 5 years not possible - CNS disease | - Cerebral, cardiovascular, and systemic condition that suggest other mechanisms for stroke - Contraindication to medical therapy - Organ failure (kidney, liver or lung) - Inability to attend follow-up | - Life expectancy <1 year - Modified Rankin Score of 3 or greater - Other potential source/disease of cardio-embolism - Contraindication to medical therapy - Anatomic criteria unfavourable for GORE ® devices | - Another cause of stroke associated with PFO - Contraindication to medical therapy or endovascular treatment - Increased bleeding risk - Inability to attend follow-up - Isolated ASD or significant left-to-right shunt | - Other identifiable mechanisms od stroke (neurological and cardiovascular) |
| Mean Follow-up period (years) | 2 | 4,1 | 5,9 | 3,2 | 5,3 | 2,8 |
| Primary Outcomes | Composite: stroke or TIA, all-cause mortality within 30 d, and death from neurologic causes between 31 d and 2 y | Composite: death, nonfatal stroke, TIA, peripheral embolism | Composite: IS or early death (closure arm: within 30 d after closure or 45 d after randomization; antithrombotic arm: within 45 d after randomization) | 2 co-primary endpoints:   - Freedom from clinical IS through 24 months - Composite of clinical IS or silent brain infarction | Stroke (fatal on nonfatal, ischemic or haemorrhagic) | Composite: stroke, vascular death, major bleeding |
| Secondary Outcomes | Major bleeding, death from any cause, stroke, TIA, and transient neurologic events of uncertain cause. | Cardiovascular death, new arrhythmias, myocardial infarction, re-hospitalization, bleeding, device-related problems | Complete closure of PFO on the 6-month follow-up TEE, absence of recurrent IS/TIA or cardiovascular death | - New brain infarction detected by MRI - Success of PFO closure and adverse events | Composite: IS, TIA, or systemic embolism  Disabling stroke, IS, cerebral hemorrhage, all death, success of PFO closure | Asymptomatic IS on follow-up MRI |
| Link-to-the-public-trial-registry | NCT00201461 | NCT00166257 | NCT00465270 | NCT00738894 | NCT00562289 | NCT01550588 |
| ASD, atrial septal defect; ASA, atrial septal aneurysm; CNS, central nervous system; IS, ischemic stroke; MRI, magnetic resonance imaging; PFO, patent foramen ovale; TEE, transesophageal echocardiography; TIA, transient ischemic attack | | | | | | |
