# Supplementary material for: Network meta-analysis on patent foramen ovale: is a stroke or atrial fibrillation worse?
Source: Neurol Sci. 2020 Nov 26;42(1):101–9. doi: 10.1007/s10072-020-04922-4 (PMC7819966; doi:10.1007/s10072-020-04922-4)
Supplement: Supplementary file 10 — (DOCX 25 kb) [file 10072_2020_4922_MOESM6_ESM.docx]

**Table S2. Events and Patients characteristics in the RCTs ***

| **Study** | **DEFENSE-PFO**  **_2018_** | | **CLOSE**  **_2017_** | | **REDUCE**  **_2017_** | | **RESPECT**  **_2017_** | | **PC Trial**  **_2013_** | | **CLOSURE I**  **_2012_** | |
| --- | --- | --- | --- | --- | --- | --- | --- | --- | --- | --- | --- | --- |
| Comparison | AMP vs MT | | MIX vs MT | | HLX/CF vs MT | | AMP vs MT | | AMP vs MT | | STF vs MT | |
|  | PFO closure | Medical Therapy | PFO closure | Antiplatelet therapy only | PFO closure | Medical Therapy | PFO closure | Medical Therapy | PFO closure | Medical Therapy | PFO closure | Medical Therapy |
| Drugs used during therapy  *A=Aspirin, C=Clopidogrel,  Cz=Cilostazol D=Dipyridamole, T=Ticlopidine, W=warfarin, dd=days, mm=months, yy=years* | A+C  (6 mm) | A or  A+C or A+Cz or W | A+C  (3 mm), then A or C or (A+D) | A or C or A+D | A+C  (3 dd), then same as MT | A or C or A+D | A+C  (1 mm),  then A (5 mm) | A or W or C or (A +D) | A  (5-6 mm) + T or C (1-6 mm) | Antiplatelet or W | A (2 yy) +C (6 mm) | A or W or both |
| No. of patients | 60 | 60 | 238 | 235 | 441 | 223 | 499 | 481 | 204 | 210^#^ | 447 | 462 |
| % data in NMA (weight) | 3.3 | | 13.3 | | 18.7 | | 27.5 | | 11.6 | | 25.5 | |
| **Patients characteristics** | | | | | | | | | | | | |
| Age in years *(standard deviation)* | 49 *(±15)* | 54 *(±12)* | 42.9 *(±10.1)* | 43.8 *(±10.5)* | 45.4 *(±9.3)* | 44.8 *(±9.6)* | 45.7  *(±9.7)* | 46.2 *(±10.0)* | 44.3  *(±10.2)* | 44.6  *(±10.1)* | 46.3  *(±9.6)* | 45.7  *(±9.1)* |
| Range (years) | 18-66 | | 16-60 | | 18-59 | | 18-60 | | <60 | | 18-60 | |
| Males (%) | 55.0 | 56.7 | 57.6 | 60.4 | 59.2 | 61.9 | 53.7 | 55.7 | 45.1 | 54.3 | 52.1 | 51.5 |
| Smoking (%) | 16.7 | 26.7 | 28.6 | 29.4 | 14.3 | 11.2 | 41.9 | 41.1 | 25.5 | 22.4 | 21.5 | 22.6 |
| Coronary artery disease (%) | NA | NA | NA | NA | NA | NA | 3.8 | 1.9 | 2.0 | 1.9 | 2.9 | 2 |
| Diabetes (%) | 10.0 | 13.3 | 1.3 | 3.8 | 4.1 | 4.5 | 6.6 | 8.5 | 2.5 | 2.9 | *NA* | *NA* |
| Dyslipidemia (%) | 30.0 | 41.7 | 12.6 | 15.3 | NA | NA | 39.3 | 40.5 | 24.5 | 29.5 | 47.4 | 40.9 |
| Hypertension (%) | 20.0 | 28.3 | 11.3 | 10.2 | 25.4 | 26.0 | 32.1 | 31.8 | 24.0 | 27.6 | 33.8 | 28.4 |
| Previous stroke (%) | NA | NA | 4.2 | 3.0 | 9.5 | 5.8 | 10.6 | 10.6 | 37.3 | 37.6 | 72.6 | 71.4 |
| Atrial septal aneurysm (%) | 8.3 | 13.3 | 24.8 | 26.4 | 20.4 | NA | 36.1 | 35.3 | 23.0 | 24.3 | 37.6 | 35.7 |
| Moderate or large shunt (%) | 51.7 | 56.7 | 75.2 | 73.6 | 81.9 | 80.1 | 49.5 | 48.0 | 70.2 | 60.9 | 55.9 | 50.0 |
| Mean follow up time (years) | 2.8 | | 5.3 | | 3.2 | | 5.9 | | 4.1 | | 2 | |
| **Events** | | | | | | | | | | | | |
| Stroke  *Relevance* | 0 | 5 | 0 | 14 | 6 | 12 | 18 | 28 | 1 | 5 | 12 | 13 |
|  | *0.00%* | *8.33%* | *0.00%* | *5.96%* | *1.36%* | *5.38%* | *3.61%* | *5.82%* | *0.49%* | *2.38%* | *2.68%* | *2.81%* |
| TIA  *Relevance* | 0 | 1 | 8 | 8 | 1 | 1 | 17 | 23 | 5 | 7 | 13 | 17 |
|  | *0.00%* | *1.67%* | *3.36%* | *3.40%* | *0.23%* | *0.45%* | *3.41%* | *4.78%* | *2.45%* | *3.33%* | *2.91%* | *3.68%* |
| Atrial Fibrillation (any events)  *Relevance* | 2 | 0 | 11^§^ | 2 | 29 | 1 | 24 | 9 | 6 | 2 | 23 | 3 |
|  | *3.33%* | *0.00%* | *4.62%* | *0.85%* | *6.58%* | *0.45%* | *4.81%* | *1.87%* | *2.94%* | *0.95%* | *5.15%* | *0.65%* |
| *(of which)*  Serious Atrial Fibrillation  *Relevance* | 1 | 0 | 0 | 0 | 10 | 1 | 7 | 4 | 2 | 2 | 6 | 3 |
|  | *1.67%* | *0.00%* | *0.72%* | *0.00%* | *2.27%* | *0.45%* | *1.40%* | *0.83%* | *0.98%* | *0.95%* | *1.34%* | *0.65%* |
| Major Bleeding  *Relevance* | 0 | 2 | 2 | 5 | 8 | 6 | 3 | 1 | 1 | 3 | 10 | 4 |
|  | *0.00%* | *3.33%* | *0.84%* | *2.13%* | *1.81%* | *2.69%* | *0.60%* | *0.21%* | *0.49%* | *1.43%* | *2.24%* | *0.87%* |
| Death (any causes)  *Relevance* | 0 | 0 | 0 | 0 | 2^A^ | 0 | 7 | 11 | 2^B^ | 0 | 2 | 4 |
|  | *0.00%* | *0.00%* | *0.00%* | *0.00%* | *0.45%* | *0.00%* | *1.40%* | *2.29%* | *0.98%* | *0.00%* | *0.45%* | *0.87%* |

* No significant difference between arms reported in each study

^#^ Eleven patients with peripheral embolism as index event.

^§^ AMP= 4 events / 138 Patients (2,90%); Other device= 7 events / 100 Patients (7,00%)

^A^ One death is a suicide

^B^ One death is not a cardiovascular event

AMP = Amplatzer Occluder device; HLX/CF = Helex/CardioForm device; MIX = Mixed devices; MT = Medical Therapy; NA= not available; RCTs = Randomized Clinical Trials; STF = Starflex device; vs = versus
